# Seroprevalence of brucellosis in small ruminants in organized and unorganized sectors of Gujarat state, India

**DOI:** 10.14202/vetworld.2018.1030-1036

**Published:** 2018-08-01

**Authors:** A. Kanani, S. Dabhi, Y. Patel, V. Chandra, O. R. Vinodh Kumar, R. Shome

**Affiliations:** 1 Office of Deputy Director of Animal Husbandry, F.M.D. Typing Scheme, Polytechnic Campus, Ambawadi, Ahmedabad - 380 015, Gujarat, India; 2 Department of Biomedical Technology, University School of Science, Gujarat University, Ahmedabad, Gujarat; 3 ICAR-National Institute of Veterinary Epidemiology and Disease Informatics (ICAR-NIVEDI), Yelahanka, Bengaluru - 560 064, Karnataka, India; 4 Division of Epidemiology, ICAR-Indian Veterinary Research Institute, Izatnagar, Bareilly - 243 122, Uttar Pradesh, India

**Keywords:** brucellosis, Gujarat, indirect enzyme-linked immunosorbent assay, Rose Bengal Plate test, seroprevalence, small ruminants

## Abstract

**Aim:**

The present study aimed to study the seroprevalence of brucellosis in small ruminants of Gujarat state, India, using Rose Bengal Plate test (RBPT) and indirect enzyme-linked immunosorbent assay (iELISA).

**Materials and Methods:**

A total of 2444 sera samples (675 sheep and 1769 goat) from unorganized sector and 1310 sera samples (861 sheep and 449 goat) from seven organized farms were collected for brucellosis screening.

**Results:**

In unorganized sector, 23.70% sheep (160/675) and 15.99% goat (283/1769) were positive by RBPT and 24.44% sheep (165/675) and 17.24% goat (305/1769) by iELISA. The organized sector samples showed higher seroprevalence in goat (7.79 %, 35/449) than sheep (4.06 %, 35/861) by RBPT. Similarly, in iELISA, goat samples showed a higher seroprevalence (9.35%, 42/449) compared to sheep (7.50%, 65/861). The diagnostic sensitivity and specificity of RBPT with ELISA were 88.69% and 99.65%, respectively, and showed a significant difference (p≤0.0001). The Chi-square analysis revealed a significant difference in seroprevalence between sectors (p≤0.01) and species (p≤0.01).

**Conclusion:**

The seroprevalence of brucellosis in small ruminants of Gujarat was investigated and showed a higher prevalence of brucellosis and warrants the implementation of proper preventive measures.

## Introduction

Small ruminant sector (sheep and goat) plays a crucial role in Indian economy which contributes annually about Rs. 24,000 million and Rs. 80,000 million to the rural and national economy, respectively [1]. Sheep and goats are considered the major source of livelihood, employment, and income to the millions of poor rural households and jobless women. According to the 19^th^ Livestock census, the sheep and goat population of Gujarat are 17.07 and 49.58 lakhs, respectively [2]. About 70% of sheep and goats are reared by small/marginal farmers and landless laborers in unorganized sectors which affords subsidiary income and food security. There is a very good demand for the small ruminant production in India, but diseases like brucellosis hamper the productivity due to the loss of milk production, abortion at late pregnancy, stillbirth, and reproduction failure and limit economic return from small ruminant production [3].

Brucellosis is one of the highly contagious zoonotic diseases of small ruminants characterized by abortion, retained placenta, infertility, orchitis, epididymitis, and rarely arthritis [4]. It occurs globally and endemic in many developing countries including India. Brucellosis is mainly caused by *Brucella melitensis* in sheep and goats, and most of the human infections are associated with the *B. melitensis* followed by *Brucella abortus* [5]. Apart from this, brucellosis has an adverse economic impact on international trade for milk, meat, and their products and severe risk to human health [6].

The national seroprevalence of brucellosis in sheep and goat was 7.9%, and 2.2%, respectively [7], and few studies have recorded the seroprevalence in small ruminants of Gujarat [8,9]. However, these studies were geographically limited to certain pockets of Gujarat and escalating information revealing increased seroprevalence in the recent times due to increased trade and rapid movement of livestock [7].

Considering the above facts, the study was carried out to assess the seroprevalence of brucellosis among the organized and unorganized sectors of small ruminant population in Gujarat, India.

## Materials and Methods

### Ethical approval and informed consent

The study was approved by the Institutional Animal Ethics Committee, ICAR- NIVEDI and the authors have taken permission from farm owners to publish data.

### Study design and sampling

**Figure-1:**
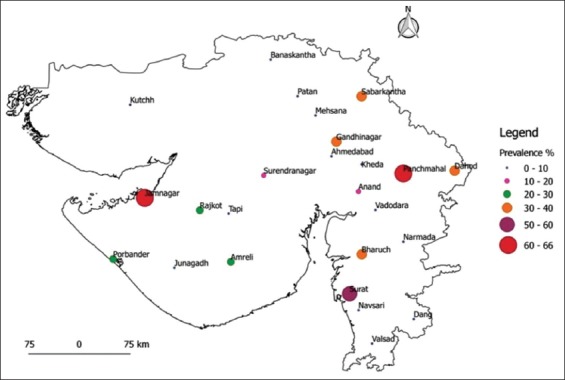
Proportion circle map showing brucellosis seroprevalence in sheep from both the sectors by indirect enzyme-linked immunosorbent assay.

A total of 3754 serum samples were collected from apparently healthy sheep and goat during 2012-2017 (5 years). Of 35 districts of Gujarat, only in 25 districts, we could gain access for the samples (Figure-1). The samples were also collected from seven organized farms of private ownership, controlled under Government Department and Board. About 2444 blood samples (675 sheep and 1769 goat) from unorganized sectors of 25 districts and 1310 blood samples (861 sheep and 449 goat) from organized farms were collected. The classification of sectors was based on a number of animals in the farm/household. In this study, the organized farms refer more than 10 number of sheep and goat in farms and unorganized refers to sheep and goat maintained by the farmers and whose number is <10. The samples were transported on ice to the laboratory and separated sera were stored at −20°C until further use. Serum samples were tested by Rose Bengal Plate test (RBPT) and indirect enzyme-linked immunosorbent assay (iELISA).

### RBPT

RBPT was performed as per the procedure described by Alton *et al*. [10]. RBPT antigen obtained from the ICAR-Indian Veterinary Research Institute, Izzatnagar, Uttar Pradesh, was used. An equal volume of 0.03 ml of serum sample and antigen was taken on the slide and mixed thoroughly. The appearance of definite clumping/agglutination within 3 min was considered a positive reaction while no clumping/agglutination as negative.

### iELISA

The iELISA developed by ICAR-National Institute of Veterinary Epidemiology, Bengaluru, for brucellosis detection was used. The test was performed according to the manufacturer’s instruction. In brief, the ELISA plate (PolySorp, Nunc) was coated with diluted sLPS antigen and incubated at 4°C overnight. Overnight incubated antigen-coated plates were washed 3 times with PBST buffer (phosphate-buffered saline pH 7.2 and Tween-20). The test and control sera were diluted in PBST (1:100) containing 2% gelatin and added to respective wells (100 µl) of the plate and incubated at 37°C for 1 h. The plates were then washed as mentioned earlier. The anti-goat/sheep IgG HRPO conjugate, diluted in PBST buffer, was added to all the wells (100 µl) and incubated for 1 h at 37°C on an orbital shaker. After washing, 100 µl of freshly prepared chromogen-substrate solution (5 mg OPD tablet in 12 ml of distilled water and 50 µl of 3% H_2_O_2_) was added and kept for color development until 10-15 min. Finally, the enzyme-substrate reaction was stopped by adding 1M H_2_SO_4_ in 50 µl volumes. The color development was read at 492 nm using an ELISA microplate reader (Tecan, Switzerland), and the results were interpreted by calculating percentage positivity (PP) as follows:


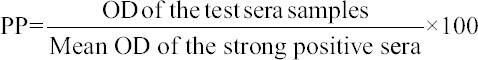


Any samples with a PP value of more than 54% were considered positive [11].

### Statistical analysis

The diagnostic statistics, namely sensitivity (Sn), specificity (Sp), positive predictive value, negative predictive value, and diagnostic accuracy, were computed [12]. The K value of <0.20, 0.21-0.40, 0.41-0.60, 0.61-0.80, and 0.81-1.0 indicated the strength of agreement as poor, fair, moderate, good, and very good, respectively. The significant difference determined by Chi-square and test value at p≤0.05 was considered statistically significant.

## Results

The seroprevalence of brucellosis in small ruminants was compared in organized versus unorganized sectors in Gujarat state. For which, a cross-sectional study was conducted during the period between 2012 and 2017 in which 675 sheep and 1769 goat samples were collected in unorganized sectors of 25 districts of Gujarat state. From seven organized farms of private ownership, a total of 1310 sera samples comprising 861 sheep and 449 goat serum sample were collected. The details of sample collected are given in Tables-1 and 2. In the unorganized sector, the seroprevalence of sheep and goat brucellosis by RBPT was 23.70% (160/675, 95% confidence interval [CI] 20.7-27.1) and 15.99% (283/1769, 95% CI 14.4-17.8) and by iELISA was 24.44% (165/675, 95% CI 21.4-27.8) and 17.24% (305/1769, 95% CI 15.6-19.1), respectively.

**Table-1:** Districtwise seroprevalence of brucellosis in sheep and goat from Gujarat.

District	Seroprevalence of sheep			Seroprevalence of goat		
	
	Total sheep samples	RBPT (% of positives)	iELISA (% of positives)	Total goat samples	RBPT (% of positives)	iELISA (% of positives)
Ahmedabad	5	0	0	95	1 (1.05)	1 (1.05)
Amreli	50	14 (28)	14 (28.00)	50	17 (34.00)	17 (34.00)
Anand	28	5 (17.86)	5 (17.86)	72	21 (29.17)	21 (29.17)
Banaskantha	0	0	0	50	0	0
Bharuch	20	8 (40.00)	8 (40.00)	80	24 (30.00)	24 (30.00)
Dahod	38	7 (18.42)	12 (31.58)	62	0	1 (1.61)
Dang	0	0	0	100	0	3 (3.00)
Gandhinagar	30	10 (33.33)	10 (33.33)	70	13 (18.57)	14 (20.00)
Jamnagar	50	33 (66.00)	33 (66.00)	50	22 (44.00)	22 (44.00)
Junagadh	50	1 (2.00)	1 (2.00)	50	2 (4.00)	2 (4.00)
Kheda	40	0	0	60	2 (3.33)	2 (3.33)
Kutch	50	2 (4.00)	2 (4.00)	50	39 (78.00)	39 (78.00)
Mehsana	15	0	0	85	23 (27.06)	23 (27.06)
Narmada	0	0	0	100	31 (31.00)	31 (31.00)
Navsari	5	0	0	95	13 (13.68)	13 (13.68)
Panchmahal	35	24 (68.57)	23 (65.71)	65	0	0
Patan	20	0	0	80	13 (16.25)	13 (16.25)
Porbandar	50	15 (30.00)	14 (28.00)	50	17 (34.00)	17 (34.00)
Rajkot	98	21 (21.43)	24 (24.49)	112	28 (25.00)	30 (26.78)
Sabarkantha	23	8 (34.78)	7 (30.43)	77	5 (6.49)	5 (6.49)
Surat	5	3 (60.00)	3 (60.00)	75	0	0
Surendranagar	53	9 (16.98)	9 (16.98)	47	5 (10.64)	7 (14.89)
Tapi	10	0	0	80	6 (7.50)	19 (23.75)
Vadodara	0	0	0	64	1 (1.56)	1 (1.56)
Valsad	0	0	0	50	0	0
Total	675	160 (23.70)	165 (24.44)	1769	283 (15.90)	305 (17.24)

The values within the parentheses indicate percentage. iELISA: Indirect enzyme-linked immunosorbent assay, RBPT=Rose Bengal Plate test

**Table-2:** Farm-wise brucellosis seropositivity in organized sheep and goat farms from Gujarat.

Farm code	Seroprevalence of sheep			Seroprevalence of goat		
	
	Total sheep samples	RBPT positives	iELISA positives	Total goat samples	RBPT positives	iELISA positives
Farm No. A	170	1 (0.59)	7 (4.12)	12	0	0
Farm No. B	140	28 (20.00)	38 (27.14)	93	3 (3.22)	9 (9.68)
Farm No. C	336	0	5 (1.49)	0	0	0
Farm No. D	147	6 (4.08)	13 (8.84)	0	0	0
Farm No. E	68	0	2 (2.94)	0	0	0
Farm No. F	0	0	0	149	32 (21.48)	32 (21.48)
Farm No. G	0	0	0	195	0	1 (0.51)
Total	861	35 (4.06)	65 (7.50)	449	35 (7.79)	42 (9.35)

The values within the parentheses indicate percentage. iELISA: Indirect enzymelinked immunosorbent assay, RBPT=Rose Bengal Plate test

**Figure-2:**
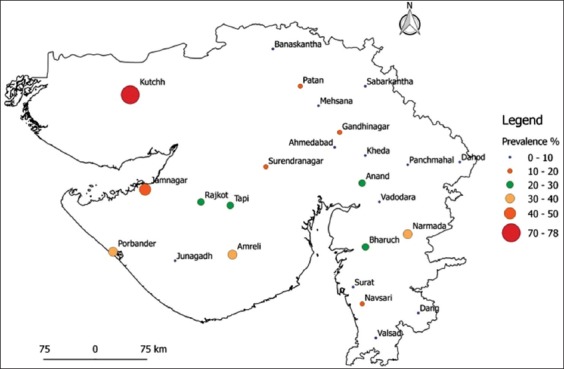
Proportion circle map for brucellosis seroprevalence in goat from both the sectors by indirect enzyme-linked immunosorbent assay.

**Figure-3:**
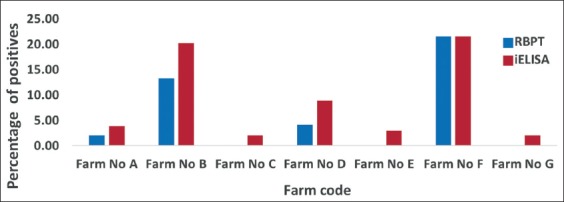
Organized farm wise seropositivity for brucellosis in small ruminants by indirect enzyme-linked immunosorbent assay and Rose Bengal Plate test.

In organized sector, the seroprevalence of sheep and goat brucellosis by RBPT was 4.06% (35/861, 95% CI 2.9-5.6) and 7.79% (35/449, 95% CI 5.7-10.7) and iELISA was 7.50% (65/861, 95% CI 6.0-9.5) and 9.35% (42/449, 95% CI 7.0-12.4), respectively. Of the 25 districts, 10 districts showed negative for sheep brucellosis, while four districts showed negative for goat brucellosis. The highest seroprevalence of sheep brucellosis was noticed in Jamnagar (66 %), while for goat, it was noticed in Kutch (78%). For goat brucellosis, Banaskantha, Panchmahal, Surat, and Valsad districts showed zero percentage seropositivity. In this study, an attempt was made to collect samples from all the districts, but due to inaccessibility and frequent migration of small ruminants, the designated numbers could not be collected in a few districts. The overall seroprevalence of brucellosis in small ruminants was 13.60% (513/3754) and 15.30% (577/3754) by RBPT and iELISA, respectively. The goats in organized sector showed comparatively higher seroprevalence (RBPT - 7.79% and iELISA - 9.35%); in contrast, sheep of unorganized sector showed higher seroprevalence (RBPT - 23.70% and iELISA - 24.44%). The proportion circle map of sheep and goat seroprevalence is shown in Figures-1-3. The RBPT and iELISA were compared for assessing the diagnostic efficiency of the tests. The diagnostic Sn and Sp of RBPT in comparison with iELISA were found to be 88.69% and 99.65%, respectively, with a Kappa value of 0.91 between the tests (Table-3). The Chi-square analysis revealed a significant difference in seroprevalence between sectors (p≤0.01) and species (p≤0.01) (Table-4).

**Table-3:** Diagnostic sensitivity and specificity of RBPT with iELISA for the detection of *Brucella* antibodies in small ruminants.

Test	iELISA		Total	Sn (%)	Sp (%)	p-value

	Positive	Negative				
RBPT						
Positive	502	11	513	88.69	99.65	p≤0.0001 (χ^2^ value=3172.47)
Negative	64	3177	3241			
Total	566	3188	3754			

Kappa value: 0.91 (95% CI: 0.90-0.93). iELISA: Indirect enzyme-linked immunosorbent assay, RBPT=Rose Bengal Plate test, CI=Confidence interval, Sn=Sensitivity, Sp=Specificity

**Table-4:** Comparison of RBPT and iELISA tests with respect to species.

Sector	Species	p-value	Sera samples tested	RBPT positives (%)	iELISA positives (%)
Unorganized	Sheep	≤0.01	675	160 (23.70)	165 (24.44)
	Goat		1769	283 (15.99)	305 (17.24)
Organized	Sheep	≤0.01	861	35 (4.06)	65 (7.50)
	Goat		449	35 (7.79)	42 (9.35)

iELISA: Indirect enzyme-linked immunosorbent assay, RBPT=Rose Bengal Plate test

## Discussion

Brucellosis is an important zoonosis which causes abortion in naturally infected small ruminants and is of great public health concern in many countries of the world [13]. In India, close contact between humans and animals is very common due to the sharing of the same housing enclosures [14]. This study provides updates on the prevalence of brucellosis in sheep and goat in the state of Gujarat. Brucellosis prevalence was recorded comparatively higher in goats in organized sector (RBPT - 7.79% and iELISA - 9.35%), whereas higher in sheep in unorganized sector (RBPT-23.70% and iELISA - 24.44%). These results were concordance with earlier reports [15,16]. Brucellosis seroprevalence in small ruminants could be associated with the frequent introduction of purchased animals into the flock, absence of quarantine/segregation, mixing of different species or infected flocks, improper disposal of aborted fetus and placental membranes, and contact of healthy animals with contaminated feed and water [16,17]. Lack of vaccination and control strategies for small ruminants may further increase the brucellosis prevalence.

To control and eradicate brucellosis from small ruminants, it is very important to establish an appropriate serological method for the diagnosis of brucellosis in the endemic areas. The diagnostic tests used may not reveal all infected animals or may give false negative results due to long incubation period, latency, or criteria used to interpret the results [18,19]. Although isolation and identification of organism are considered gold standard for diagnosis, it is cumbersome, takes several days to weeks, and poses a greater risk to laboratory personnel. Hence, the diagnosis of brucellosis largely depends on the use of two or more tests to confirm any positive animals [20,21]. RBPT is a screening test, and iELISA is a confirmatory test used for *B. melitensis* infection in sheep and goats [19,21,22]**.** In comparison to iELISA, the Sn and Sp of the RBPT are low. However, in view of cost, feasibility, and reliability as a field diagnostic test, RBPT has been found to be convenient to perform than the iELISA [9].

The study revealed the overall seroprevalence of brucellosis in small ruminants as 13.60% (513/3754) and 15.30% (577/3754) by RBPT and iELISA, respectively. The seroprevalence was recorded in 23 of 25 districts screened from Gujarat state. Higher seroprevalence by iELISA in sheep was observed in Jamnagar, Panchmahal, Surat, Bharuch, Gandhinagar, and Sabarkanth, whereas, in goats, higher seroprevalence was recorded from Kutch, Jamnagar, Amreli, Porbandar, Narmada, and Bharuch districts. Only two districts (Kutch and Jamnagar) have shown higher seroprevalence of brucellosis in both sheep and goat indicating either endemicity/hotspot for brucellosis. Low prevalence was observed in Ahmedabad, Dang, Junagadh, Kheda, and Vadodara districts. Anti-*brucella* antibody was not recorded in the goat samples screened from Banaskantha, Panchmahal, Surat, and Valsad. We could not attribute any reason for zero prevalence of goat brucellosis in these four districts.

The present seroprevalence rate is lower than the seroprevalence report of Shome *et al*. [11], who have reported 26.08% and 22.60% seropositivity by RBPT and iELISA, respectively. It could due to more number of samples or wider geographical area covered in the current study. Among serological tests, the highest seropositivity was recorded by iELISA (17.13%) than RBPT (13.67%). Similar results have been reported [23-26]. In contrast, few authors have reported lower seropositivity by iELISA compared to the RBPT [17,23,27]. The iELISA kit was procured from ICAR-NIVEDI for screening brucellosis in Gujarat (Indian Patent No. 250709). For comparison purpose, routinely used RBPT was carried out with the indigenously procured iELISA kit. Since the kit has shown a kappa value of 0.90-0.93, it reflects the performance of the kit as excellent. This indirectly verifies the correct assessment of seroprevalence of brucellosis in the study and the use of diagnostic assays either RBPT or iELISA or both for screening farms or surveillance as per the availability of facilities.

Seroprevalence of brucellosis in small ruminants was higher in unorganized sector (18.10% [443/2444) for RBPT and 19.20% [470/2444] for iELISA) compared to the organized sector (5.30% [70/1310] for RBPT and 8.10% [107/1310] for iELISA). The results were similar to the Sharma *et al*. [26] and contrary to few reports [25,28,29]. The low prevalence of brucellosis in the organized sector could be due to better management practices and routine screening of animals for brucellosis. Vaccination against brucellosis is one of the most effective measures to reduce the prevalence of disease and has largely contributed to the success of control programs in many countries [30]. Test and slaughter policy routinely recommended in brucellosis eradication program only when the prevalence of infected animals is 2% or below and the flocks are maintained under closely controlled conditions and protected efficiently against reentry of infection [20]. However, it is not possible in developing countries like India. Hence, selective culling of high-level shedders may be an effective and feasible alternative to a comprehensive test and slaughter program [31].

## Conclusion

The results of the present study indicate the higher prevalence of brucellosis among the small ruminant’s population of Gujarat, India. Furthermore, they may act as a potential public health hazard for the spread of brucellosis to humans as well as other animals due to their stay in a close association with the human community. Hence, it warrants the formulation of control measures, routine screening, and mass vaccination for small ruminants along with the public awareness programs to reduce the zoonotic risk among the human population.

## Authors’ Contributions

The present study was a part of AICRP-ADMAS project work. AK, SD, YP, and VC planned the study, collected the samples, conducted the experiments, and entered the data for analysis. OR VK and RS carried out statistical analysis, interpretation of the results, and drafting and finalizing the manuscript. All authors read and approved the final manuscript.

## References

[1] Sen A.R, Santra A, Karim S.A (2004). Carcass yield, composition and meat quality attributes of sheep and goat under semiarid conditions. Meat Sci.

[2] Livestock Census (2012). All India Report, Ministry of Agriculture Department of Animal Husbandry.

[3] Megid J, Mathias L.A, Robles C.A (2010). Clinical manifestations of brucellosis in domestic animals and humans. Open Vet. Sci. J.

[4] Radostits O.M, Gay C, Blood C.D, Hinchclift W.K (2007). Veterinary Medicine, Textbook of the Diseases of Cattle, Sheep, Pigs, Goats and Horses.

[5] Mantur B.G, Amarnath S.K (2008). Brucellosis in India–a review. J. Biosci.

[6] Gul S, Khan A (2007). Epidemiology and epizootiology of brucellosis: A review. Pak. Vet. J.

[7] Renukaradhya G.J, Isloor S, Rajasekhar M (2002). Epidemiology, zoonotic aspects, vaccination and control/eradication of brucellosis in India. Vet. Microbiol.

[8] Patel M.D, Patel P.R, Prajapati M.G, Kanani A.N, Tyagi K.K, Fulsoundar A.B (2014). Prevalence and risk factor's analysis of bovine brucellosis in peri-urban areas under intensive system of production in Gujarat, India. Vet. World.

[9] Padher R.R, Nayak J.B, Brahmbhatt M.N, Mathakiya R.A (2017). Comparative sensitivity and specificity of various serological tests for detection of brucellosis in small ruminants. Int. J. Curr. Microbiol. App. Sci.

[10] Alton G.G, Jones L.M, Angus R.D, Verger J.M (1988). Techniques for Brucellosis. Institute National de le RecherchéAgronomique.

[11] Shome R, Shome B.R, Deivanai M, Desai G.S, Patil S.S, Bhure S.K, Prabhudas K (2006). Seroprevalence of brucellosis in small ruminants. Indian J. Comp. Microbiol. Immunol. Infect. Dis.

[12] Thrusfield M (2013). Veterinary Epidemiology.

[13] Benkirane A, Essamkaoui S, Idrissi E.L, Lucchese L, Natale A (2015). A serosurvey of major infectious causes of abortion in small ruminants in Morocco. Vet. Ital.

[14] Ashenafi F, Teshale S, Ejeta G, Fikru R, Laikemariam Y (2007). Distribution of brucellosis among small ruminants in the pastoral region of Afar, Eastern Ethiopia. Rev. Sci. Tech.

[15] Singh S.V, Agarwal G.S, Batra H.V, Gupta V.K, Singh N (2000). Monitoring of *Brucella* infection associated with reproductive losses using multiple serological tests in organized goat and sheep flocks. Indian J. Anim. Sci.

[16] Sadhu D.B, Panchasara H.H, Chauhan H.C, Sutariya D.R, Parmar V.L, Prajapati H.B (2015). Seroprevalence and comparison of different serological tests for brucellosis detection in small ruminants. Vet. World.

[17] Sharifi H, Mashayekhi K, Tavakoli M.M (2015). Risk factors of small ruminant brucellosis: A cross-sectional study in Southeast Iran 2012. Hum. Vet. Med. Int. J. Bioflux Soc.

[18] Kolar J (1984). Diagnosis and control of brucellosis in small ruminants. Prev. Vet. Med.

[19] Nicoletti P (1993). The eradication of brucellosis in animals. Saudi Med. J.

[20] Mahajan N.K, Kulshreshtha R.C (1991). Comparison of serological tests for *Brucella melitensis* infection in sheep. Trop. Anim Health Prod.

[21] Radulescu R.A, Petriceanu G, Ragalie A, Gutu E (2007). Comparative evaluation of serological assays for brucellosis diagnosis. Rev. Rom. Med. Vet.

[22] OIE Manual of Standards for Diagnostic Tests and Vaccines for Terrestrial Animals (2017). Ch. 2.1.4. Brucellosis (Brucella abortus, B. melitensis and B. suis) (infection with B. abortus, B. melitensis and B. suis) (NB: Version Adopted in May 2016).

[23] Kotadiya A.J (2012). Serological, Cultural and Molecular Detection of *Brucella* Infection of Sheep in Gujarat. M. V. Sc. Thesis Submitted to Sardarkrushinagar Dantiwada Agriculture University, Sardarkrushinagar, Gujarat.

[24] Reddy D.A, Kumari G, Rajagunalan S, Singh D.K, Kumar A, Kumar P (2014). Seroprevalence of caprine brucellosis in Karnataka. Vet. World.

[25] Sharma P, Kotwal S.K, Singh M, Sharma H.K (2015). Comparative serological study on antibodies against *Brucella* in small ruminants. Indian Vet. J.

[26] Sharma V, Sharma H.K, Ganguly S, Berian S, Malik M.A (2017). Seroprevalence studies of brucellosis among goats using different serological tests. J. Entom. Zool. Stud.

[27] Rahman M.S, Ali Hahsin M.F, Ahasan M.S, Her M, Kim J.Y, Kang S, Jung S.C (2011). Brucellosis in sheep and goat of Bogra and Mymensingh districts of Bangladesh. Korean J. Vet. Res.

[28] Singh A, Agrawal R, Singh R, Singh D.K, Pande N (2010). Seroprevalence of brucellosis in small ruminants. Indian Vet. J.

[29] Sharma V.K, Savalia C.V, Selvam D.T, Darekar S.D (2006). Seroprevalence of caprine and ovine brucellosis in Mehsana and Patan districts of Gujarat. Intas-Polivet.

[30] Hasannia E, Soleimani S, Alamian S, Behrozikhah A, Emadi A, Dostdari S (2015). Stability Study of Iriba brucellosis full-dose and reduced-dose vaccine produced by Razi Institute in Iran. Arch. Razi Instit.

[31] Higgins J.L, Gonzalez-Juarrero M, Bowen R.A (2017). Evaluation of shedding, tissue burdens and humoral immune response in goats after experimental challenge with the virulent *Brucella melitensis* strain 16M and the reduced virulence vaccine strain. Rev. 1. PLoS One.

